# Bilateral Arteriovenous Shunting Through Pial and Perforating Vessels With Multiple Strokes in a Patient With Hepatopulmonary Syndrome

**DOI:** 10.7759/cureus.42756

**Published:** 2023-07-31

**Authors:** Baha Aljeradat, Manisha Koneru, Renato Oliveira, Hamza Shaikh

**Affiliations:** 1 Neurosurgery, The University of Jordan School of Medicine, Amman, JOR; 2 Neurosurgery, Cooper Medical School of Rowan University, Camden, USA; 3 Neurointerventional Surgery, Cooper University Health Care, Camden, USA

**Keywords:** cerebral proliferative angiopathy, cerebral amyloid angiopathy, intracerebral haemorrhage, cerebro-vascular accident (stroke), hepatopulmonary syndrome, cerebral arteriovenous shunting

## Abstract

Hepatopulmonary syndrome (HPS) is a condition characterized by chronic liver disease, intrapulmonary arteriovenous shunting, and increased alveolar-arterial oxygen gradient. This case report presents a 54-year-old male patient with a history of stroke, liver cirrhosis, portal vein thrombosis, hypertension, diabetes, and bladder cancer, who presented with worsening headaches and confusion over the course of five years. Digital subtraction angiogram (DSA) revealed multiple bilateral arteriovenous shunts, suggesting a shunting mechanism similar to that observed in HPS. We propose that this unique case could provide valuable insights into the parallels between the pathophysiology of HPS and diffuse arteriovenous shunting in the brain and the increased risk of ischemic and hemorrhagic events in both cases. Further studies are needed to establish a clearer understanding of this relationship and its implications for patients with chronic liver disease.

## Introduction

A diffuse cerebral proliferative angiopathy (CPA), previously known as diffuse arteriovenous malformation (AVM), is a rare cerebrovascular malformation affecting the brain, characterized by intricate vascular structures that infiltrate the brain tissue. Typically, this disorder is diagnosed in the context of hereditary syndromes presenting with hemorrhages or seizures. CPAs are usually fed by arteries originating from both the anterior and posterior circulation. A bilateral arterial supply is not uncommon in CPAs. The angiographic appearance of CPAs includes multiple feeding arteries, lack of typical AVM nidus, and large, tortious draining veins, with suggestions that neural tissue may be present within the malformation itself [1-5]. An example of this condition was shown in a recent case study that reported a 20-year-old man who presented with ptosis of the left eyelid. Radiological imaging revealed a CPA consisting of multiple arteriovenous shunts that drained bilaterally to both hemispheres through several dilated veins but lacked the typical arteriovenous malformation nidus [1]. In a study of 1434 patients with brain AVMs, a group of 49 patients was identified to have distinct angiographic characteristics suggesting CPAs. Symptomatically, this subgroup presented with seizures, disabling headaches, and stroke-like symptoms, while hemorrhagic presentations were less common [6].

Hepatopulmonary syndrome (HPS) is a condition that is characterized by three main features: chronic liver disease, the presence of intrapulmonary arteriovenous shunting, and increased alveolar-arterial oxygen gradient. In HPS, the distal pulmonary arteries enlarge and become twisted, resulting in the shunting of blood and subsequent AVM formation. HPS presents clinically with gradual onset of difficulty breathing, bluish skin coloration, and nail clubbing in individuals with a medical history of chronic liver disease, such as cirrhosis. Chest radiographs and computed tomography (CT) often display nodular or reticulonodular opacities in the lower lung fields, which are typically caused by the dilation of lung vessels. Pulmonary angiography in HPS patients reveals distal arterial dilation and arteriovenous shunting [7-12].

An association between hepatovascular system abnormalities and cerebrovascular pathologies has been noted in the literature. For example, patients with hepatitis C infection are more likely to have strokes; cirrhotic patients are at increased risk for hemorrhagic stroke. A prior case report described the aberrant proliferation of small perforating cerebral arteries, in the absence of classical AVM, in the setting of hepatopulmonary syndrome causing multifocal ischemic stroke and intracerebral hemorrhages [12-15].

Our patient with multiple strokes due to diffuse intracranial shunting in the setting of HPS intends to highlight a potential association and a shared pathophysiologic process underlying vascular malformations both hepatically and intracranially. This case was presented as a poster at the 20th Annual Meeting of the Society of NeuroInterventional Surgery on July 31, 2023.

## Case presentation

We present a case of a 54-year-old male patient with prior history of stroke, liver cirrhosis, portal vein thrombosis, hypertension, diabetes mellitus, and bladder cancer. He has a history of several minor hemorrhagic strokes over the past five years (which we believe are of similar etiology to his current presentation); he has had progressive memory issues gradually worsening over the past two years. At baseline, he has waxing and waning awareness, ranging from speaking simple sentences to only repeating single words.

One week prior to the current presentation, he was found to have a new hemorrhagic stroke after worsening mentation and headaches. Although he was discharged after returning to baseline, he acutely worsened again and returned to the emergency department (ED). At the ED, his vitals were notable only for elevated blood pressure. Physical exam showed that the patient was awake, alert, and oriented to person only. He was agitated and not directly answering questions, although his speech was clear. He was moving his limbs spontaneously, shouting intermittently, but not following commands. The remainder of the neurologic exam was within normal limits. Point-of-care electroencephalogram (EEG) was unremarkable.

**Figure 1:**
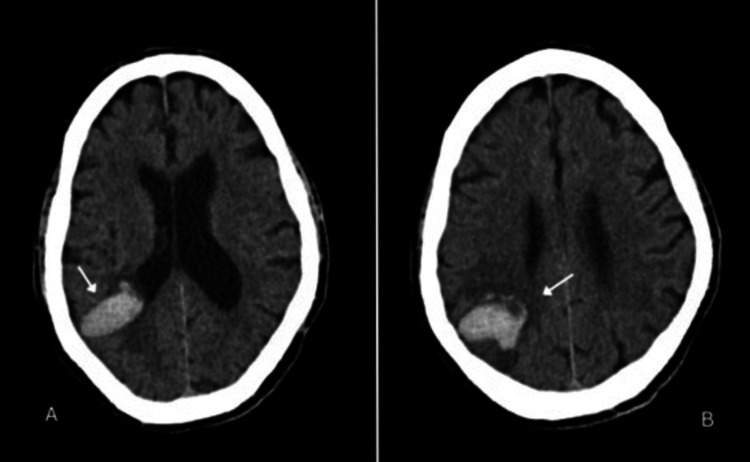
Computed tomography without contrast Axial non-contrast computed tomography (CT) scans of the head (A, B) demonstrating a 5.2 cm right parietal intraparenchymal hemorrhage with surrounding edema (arrow) and a 3 mm leftward midline shift. There is an additional right temporal intraparenchymal hemorrhage measuring 1.0 cm.

Follow-up CT head scan (Figure 1) showed a 5.2 cm non-specific right parietal hemorrhage, which had increased in size compared to one week prior, associated with edema and localized mass effect. There was a 3 mm leftward midline shift. The scan also showed a 1 cm right temporal hemorrhage with cerebellar and cerebral atrophy.

**Figure 2:**
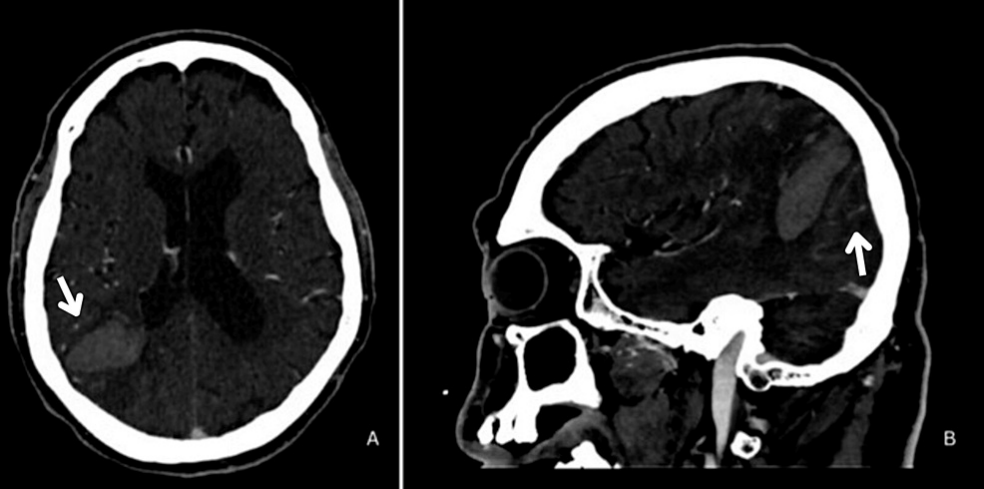
Computed tomography angiography with contrast Axial (A) and sagittal (B) computed tomography angiography (CTA) with venous contamination of the head and neck demonstrating displacement of the vascular structures surrounding the right parietal intraparenchymal hematoma due to mass effect (white arrows).

**Figure 3:**
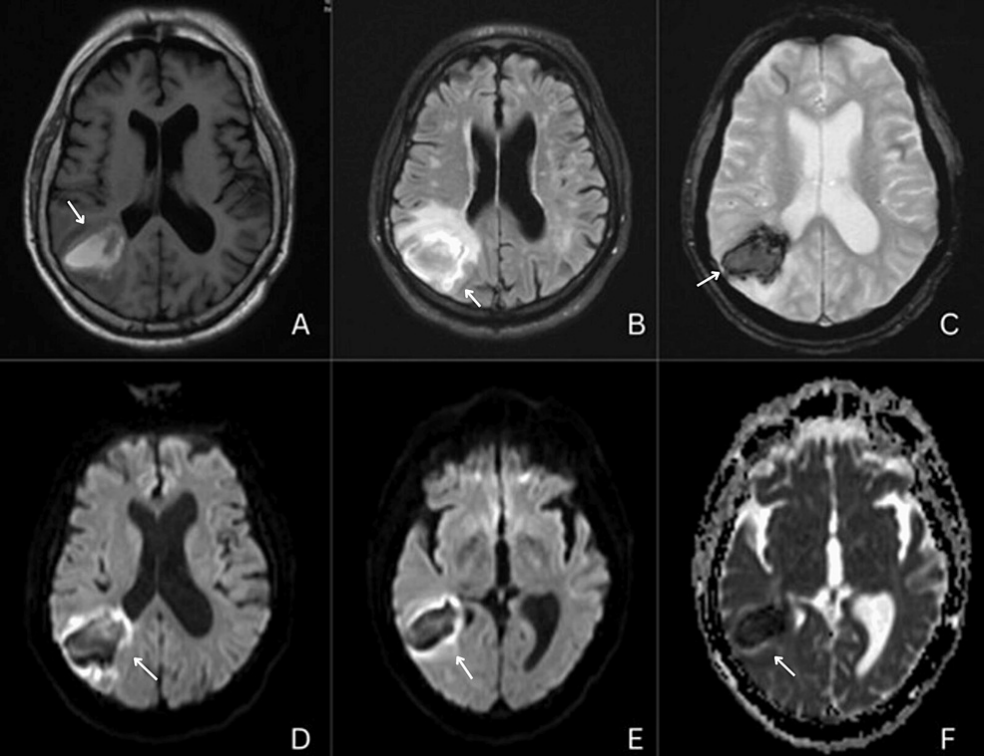
Different MRI sequences showing the right intraparenchymal hemorrhage A: T1; B: Fluid Attenuation Inversion Recovery (FLAIR); C: Gradient Echo Sequence (GRE); D, E: Diffusion Weighted Imaging (DWI); F: Apparent Diffusion Coefficient (ADC). (White arrows show the right intraparenchymal hemorrhage in the above images).

**Figure 4:**
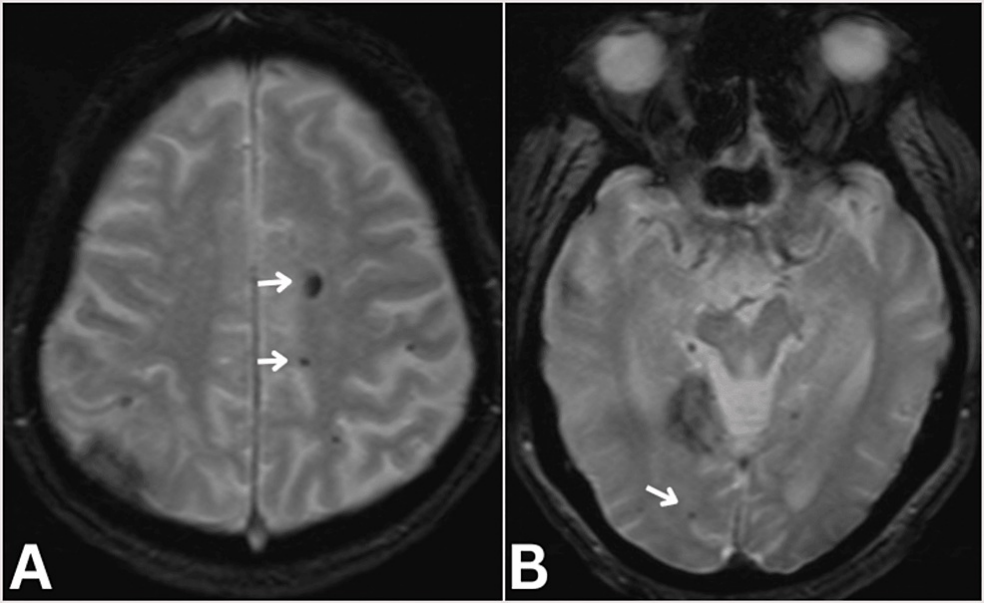
Brain GRE MRI Brain Gradient-echo GRE MRI (axial) showing diffuse microbleeds (arrows).

CT angiogram (CTA) of the head and neck (Figure 2) showed displaced vasculature due to localized mass effect. Subsequent brain magnetic resonance imaging (MRI) (Figure 3-4) showed an acute right parietal mass with local mass effect, smaller sub-acute right temporal parenchymal hemorrhage, and multiple chronic hemorrhages primarily in the right cerebellar hemisphere.

**Figure 5:**
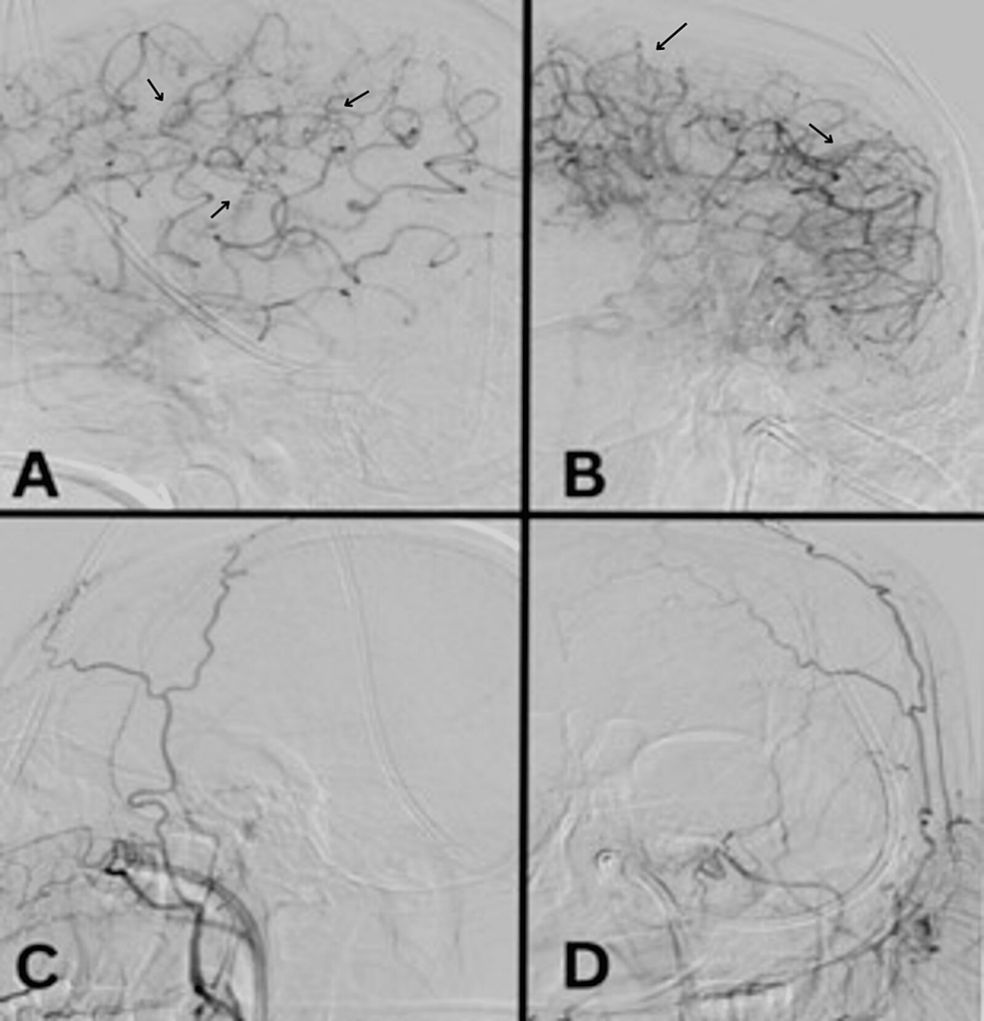
Digital subtraction angiography for left side cerebral vasculature. Lateral (A) and anteroposterior (AP) (B) views of the left internal carotid artery injection demonstrating middle cerebral artery to anterior cerebral artery pial-pial collateral flow (black arrows). Lateral (C) and AP (D) views of the left external carotid artery injection showing a lack of extracranial to intracranial anastomosis.

**Figure 6:**
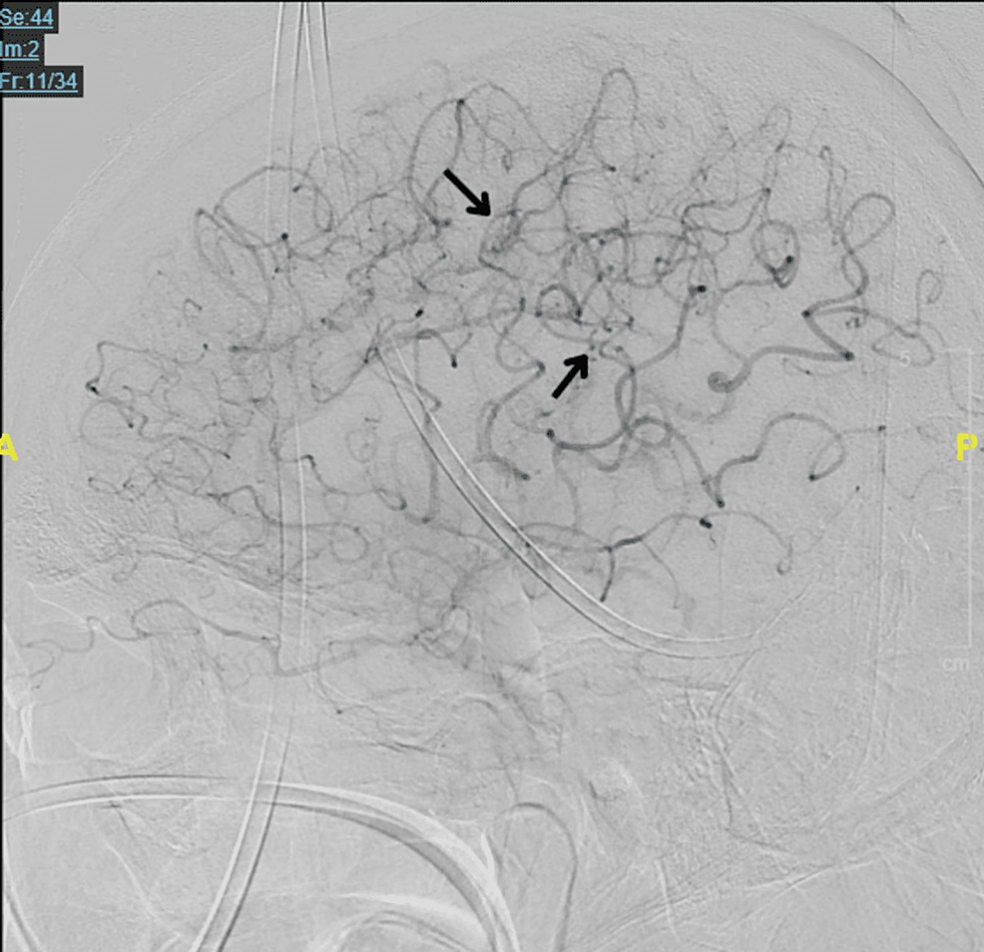
Digital subtraction angiography for the left internal carotid artery. Lateral view of the left internal carotid artery injection demonstrating middle cerebral artery to anterior cerebral artery pial-pial collateral flow (black arrows).

**Figure 7:**
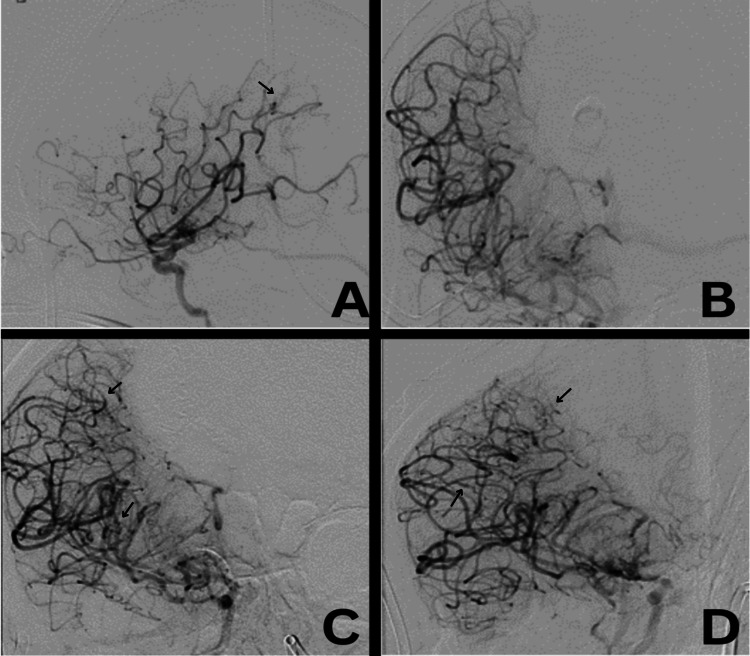
Digital subtraction angiography for right side cerebral vasculature. A: Lateral, B and C: anteroposterior (AP), D: right anterior oblique (RAO) views of arterial phase of the right internal carotid artery injections showing multiple curvilinear, irregular pial vessels adjacent to the distal middle cerebral artery with associated early arteriovenous shunting (black arrows).

**Figure 8:**
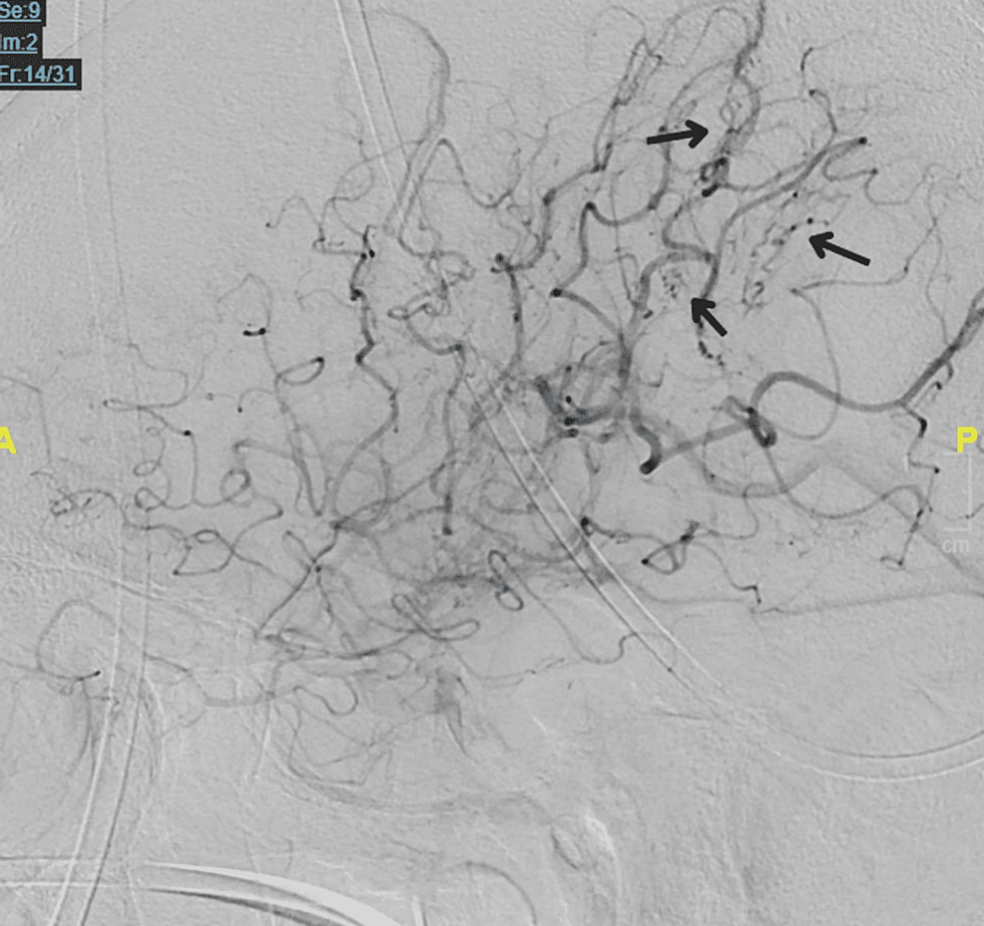
Digital subtraction angiography for right internal carotid artery Lateral view of the right internal carotid artery showing multiple curvilinear, irregular pial vessels adjacent to the distal middle cerebral artery with associated early arteriovenous shunting (black arrows).

Given the patient’s young age and multiple occurrences of hemorrhages across several locations, the possibility of a potential occult vasculopathy was considered. To evaluate for vasculopathy, the patient underwent a digital subtraction angiogram (DSA) (Figures 5-8). The DSA has shown bilateral, multiple, curvilinear, irregular pial vessels associated with early high flow arteriovenous shunting. It also showed hypertrophied lenticuloustriate. These findings were most consistent with vascular shunting from underlying altered hemodynamics, likely secondary to liver cirrhosis.

Consequently, cerebrovascular malformation conditions, such as CPAs and other reactive vasoproliferative processes, were considered. The nature of the patient's bleeds, particularly the multiple areas of cortical bleeding of different ages compounded by cirrhosis-related coagulopathy from comorbid cirrhosis, has increased the suspicion of a possible cerebral amyloid angiopathy (CAA) etiology.

To evaluate for CAA etiology, a potential brain biopsy was discussed amongst the multidisciplinary team. In the interim, further medical management of cirrhosis was pursued. Typically, anticoagulation would be pursued due to systemic coagulopathy and portal vein thrombosis history, but it was contraindicated due to hemorrhage. Consequently, supportive measures were pursued.

After DSA, the patient demonstrated improved mentation throughout the duration of his hospital course. He continued to have intermittent headaches at the time of discharge and was scheduled for a follow-up in the outpatient clinic to plan for a brain biopsy to prognosticate CAA and liver transparent candidacy.

## Discussion

This is one of the first few reported cases in which a patient with chronic liver disease has diffuse intracranial shunting similar to shunting seen in hepatopulmonary syndrome. In HPS, angiographic studies commonly show arteriovenous shunting and distal artery dilation, causing hypoxia from ventilation-perfusion mismatch [1]. This radiographic finding is similar to the angiographic appearance of our patient’s brain vessels. Similar to the adaptive hemodynamic changes seen in HPS, our patient’s intracranial shunting is likely the precipitating factor of the sequelae from the cerebrovascular hemodynamic changes in our patient [1]. Thus, this case illustrates the potential association between altered systemic hemodynamics from liver cirrhosis and diffuse arteriovenous brain shunting, paralleling the pathophysiology of HPS shunts and providing an explanation for the ischemic and hemorrhagic sequelae.

Multiple studies have described the relationship between strokes in patients with liver cirrhosis due to altered coagulability. For example, a study that compared 118 patients with liver cirrhosis to 236 controls found that cirrhotic patients had altered platelets and coagulation [15,16]. In several systematic reviews and meta-analyses, there was a variation in the incidence of strokes in cirrhotic patients. However, the analyses found an increased risk of hemorrhagic strokes over ischemic strokes in this comorbid population [17,18]. In our patient, we saw multiple hemorrhagic incidents that align with the literature that suggests an increased risk of hemorrhage in liver cirrhosis patients.

The differential for our patient included several cerebrovascular proliferative diseases, including AVMs and diffuse CPAs. Ultimately, clinical course and comorbid conditions raised suspicion for CAA. CAA is characterized by amyloid-beta deposits in cortical and leptomeningeal blood vessels, and this condition is associated with multiple recurrent intracranial bleeds, progressive cognitive decline, and other neurological abnormalities [19]. Brain biopsy is the modality to assess the extent of amyloid deposition. In this case, a brain biopsy was pursued to assess the extent of the disease and to prognosticate for a potential liver transplant. In intracranial arteriovenous shunting states, it has been shown that aggressive management of aberrant liver pathology has a positive effect on intracranial shunting. Prior reported cases describe the spontaneous disappearance of intracranial arteriovenous malformation after living-donor liver transplantation. Resolution of intracranial pathology following the resolution of liver pathology potentially supports the hypothesis of the association between chronic liver disease and cerebral arteriovenous shunting [20].

Future studies can better characterize the pathophysiology and risk factors modifying the relationship between liver cirrhosis and diffuse arteriovenous shunting and its role in precipitating the risk of ischemic and hemorrhagic events, particularly in the presence of more atypical arteriovenous malformation pathology.

## Conclusions

AVMs occur in the brain and present with intracranial shunting dynamics similar to that seen in HPS, a consequence of chronic liver disease. Similar pathophysiology driving HPS may potentially be systemically affecting cerebrovascular hemodynamics in cirrhotic patients, thus precipitating atypical arteriovenous shunting conditions, such as CPAs and CAAs. Moreover, altered hemodynamics and coagulopathic state may increase the risk for hemorrhagic stroke in this population. This potential relationship was evidenced in our patient, whose systemic complications from long-term cirrhosis may have also encompassed his gradual confusion, multiple hemorrhagic cerebrovascular accidents, intracranial shunting, and CAA pathology.

This case provides insight into the parallel pathophysiology of HPS and diffuse arteriovenous shunting in the brain and the consequential risk of hemorrhagic events in systemically altered hemodynamic states. Further studies can be performed to better examine the relationship between hepatic and cerebrovascular pathology to elucidate potential implications for the long-term management of patients with chronic liver disease.
